# The Relationship between Perceived Coaching Behaviors, Competitive Trait Anxiety, and Athlete Burnout: A Cross-Sectional Study

**DOI:** 10.3390/ijerph16081424

**Published:** 2019-04-21

**Authors:** Seongkwan Cho, Hunhyuk Choi, Youngsook Kim

**Affiliations:** 1 College of Nursing and Health Sciences, Texas A&M International University, Laredo, TX 78045, USA; scho@tamiu.edu; 2 Department of Physical Education, Korea National University of Education, Cheongju 28173, Korea; dkheon@gmail.com; 3 Department of Sports Science, Korea Institute of Sport Science, Seoul 01794, Korea

**Keywords:** perceived coaching behaviors, autonomy-supportive coaching, controlling coaching, trait anxiety, athlete burnout

## Abstract

Athletes possibly experience a great deal of stress which may cause anxiety and burnout. Athletes’ perceptions of their coaches’ behaviors influence their performance and psychological well-being. The purpose of this study is to investigate the relationship between athletes’ perception of their coaches’ coaching behaviors and burnout, and also to examine the medication effects of competitive trait anxiety on the relationship. A total of 368 collegiate athletes participated in the study, and their ages ranged from 20 to 26 years old (*M*_age_ = 21.21 years, *SD* = 1.07 years). A cross-sectional research design was employed to collect the data. Descriptive statistics and structural equation modeling are utilized to analyze the data. Trait anxiety in athletes had a significant correlation with athlete burnout as well as significant pathways. Controlling coaching behaviors were significantly related to athletes’ competitive trait anxiety, whereas autonomy-supportive coaching behaviors were not significantly related to trait anxiety. A significant positive pathway from controlling coaching to trait anxiety was observed. The bootstrapping results indicated a significant and indirect pathway from controlling coaching to athlete burnout via competitive trait anxiety. Given that controlling coaching behaviors affected trait anxiety and, in turn, burnout, it is concluded that coaches should provide less controlling coaching to reduce anxiety and burnout in athletes.

## 1. Introduction

Sport environments can provide excessive stress for athletes, and long-term exposure to the stress may cause burnout [1,2]. Raedeke [3] first proposed the multidimensional construct of burnout by adopting Maslach and Jackson’s definition of burnout (i.e., a psychological syndrome of emotional exhaustion, depersonalization, and reduced personal accomplishment) [4]. However, because depersonalization in Maslach and Jackson’s definition is negative feelings and reactions towards clients and is less applicable to athletes, Raedeke [3] replaced depersonalization with sport devaluation in order to explain athletes’ negative feelings and attitudes toward their sports and defined athlete burnout as a syndrome composed of emotional and physical exhaustion, sport devaluation, and a reduced sense of accomplishment. Due to the lack of a valid and reliable questionnaire to measure burnout in athletes, Raedeke and Smith [5] later developed a sport-specific burnout questionnaire (i.e., Athlete Burnout Questionnaire; ABQ). The development of the ABQ advanced burnout research in the athlete population [6]. Early signs of burnout include emotional and physical tiredness or fatigue, mood disturbance, lack of enjoyment, loss of motivation, and perceptions of inadequate social support [3,7]. Smith [8] proposed four stages of the cognitive-affective model to better understand athlete burnout. The first stage is related to “interactions between the environmental demands and personal and environmental resources” [8] (p. 41). High competitive demands, low social support, and low autonomy can increase or decrease the environmental demands. In the cognitive appraisal stage, each athlete appraises the situational demands unequally. The imbalance between demands and resources causes stress. The athlete shows physiological responses (e.g., tension, anxiety, depression, and fatigue) when perceiving the demand as threatening. The physiological responses lead athletes to the last stage, coping and task behaviors, including decreased performance and withdrawal from activities. Within the cognitive-affective burnout model, withdrawal from sports would be one of the behavioral consequences, but burnout is not “the primary cause of sport withdrawal” [9] (p. 277). Recent studies in various settings showed work-related stress was a significant predictor of burnout [10,11,12], and studies in sport-specific settings also provided supportive results that chronic stress is highly related to burnout [9,13,14,15,16].

Anxiety is a reaction by an individual to a stressful situation [17], and athletes in competitive sports possibly have a great deal of performance-related stress. In early studies, researchers modified and used general anxiety measures to examine anxiety in sports, but they found sport-specific anxiety measures to be better predictors of athletes’ behavior. For example, the Sport Anxiety Scale-2 has been used to examine the multidimensional trait anxiety in sport contexts [18]. Theses sport-specific anxiety measures have helped researchers to obtain valid and reliable data in order to investigate the effects of anxiety on athletic performance, injury, and burnout in athletes.

The relationship between anxiety and burnout was predictable from Smith’s cognitive-affective model of athlete burnout [8]. As mentioned in the model, burnout is related to a complex cognitive process and is a consequence of chronic stress which can be intimately related to anxiety, especially cognitive anxiety. Studies have supported that anxiety would be one of the predictors of burnout in athletes [14,15,16,19]. Athletes reported feelings of frustration, lack of confidence, and concentration problems as mental symptoms of burnout that could also be interpreted as cognitive anxiety [13]. Trait anxiety as a dispositional characteristic was the best predictor of burnout among all intrapersonal and situational predictors, and the cognitive appraisal of and physiological responses to stress could influence the development of burnout [16]. Cremades et al. [19] also found several significant correlations between trait anxiety and burnout in collegiate athletes. The higher the levels of trait anxiety, the more risk an athlete has of becoming burned out [20].

There are diverse sources of stress for athletes, and identifying and understanding them helps to understand the development and prevention of anxiety as well as burnout in athletes. One of the potential sources can be coaches’ behaviors as an interpersonal factor. In sports, coaches play an influential role in affecting anxiety in athletes [21], and previous studies found a significant relationship between perceived coaching behaviors and anxiety. Gould and Weinberg [22] suggest the use of the interaction approach, which includes interpersonal and situational factors to understand anxiety and burnout in competitive sport environments. Ryska and Yin [23] examined the relationship between the athlete’s perceptions of coach support and precompetitive anxiety in high school athletes and found high coach support lowered precompetitive anxiety. Baker, Côté, and Hawes [24] have highlighted that negative perceptions of coaching behaviors had a positive correlation with trait anxiety levels in athletes. Baker et al. also suggested levels of anxiety in athletes would increase if a negative relationship between a coach and athlete exists. Studies also found a significant relationship between perceived coaching behaviors and burnout. Autonomy-supportive coaching behaviors were negatively related to athlete burnout, whereas controlling coaching behaviors were positively related to athlete burnout [25]. Less controlling and more autonomy coaching behaviors might lower levels of burnout or avoid the development of burnout in elite athletes [25,26]. Athletes with more autonomy-supportive environments also felt less anxious and burned-out [27]. Recent studies in non-sport settings also showed consistent results that perceptions of leaders’ and supervisors’ support significantly predicted burnout [11,28]. Vealey and colleagues [15] examined the effect of athletes’ perceptions of coaching behaviors on burnout and competitive anxiety and also tested the relationship between athletes’ levels of competitive (trait) anxiety and burnout. All competitive trait anxiety subscales (i.e., somatic anxiety, worry, and concentration disruption) significantly predicted burnout in athletes. Somatic anxiety was a weaker predictor of burnout than cognitive anxiety (i.e., worry and concentration destruction). 

In sum, from the results of previous research, some correlations and causal relationships were revealed between perceived coaching behaviors, anxiety, and perfectionism. However, these results were inconsistent. For example, although Vealey et al. [8] did not find any relationship between perceived coaching behaviors and anxiety, they found that perceived coaching behaviors significantly influenced anxiety [23,24]. The inconsistent findings have required further research; however, there was no study investigating causal relationships. Therefore, the purpose of this study was simultaneously to examine the mediating effects of competitive trait anxiety on the relationship between perceived coaching behaviors and athlete burnout. 

## 2. Materials and Methods

### 2.1. Participants

A total of 368 collegiate athletes (288 males and 80 females) from 12 colleges in Korea participated in the study. The subjects participated in various sports, including track and field (17 males and 10 females), weightlifting (16 males), swimming (28 males and 14 females), gymnastics (17 males and 1 females), golf (21 males and 15 females), basketball (35 males and 10 females), taekwondo (51 males and 16 females), baseball (35 males), judo (27 males and 14 females), and soccer (41 males). The participants’ ages ranged from 20 to 26 years old, and the mean age was 21.21 years with *SD* = 1.07 years. The participants were in pre-season. Of the participants, 27 athletes reported that they would not play due to injuries. General characteristics of the participants in this study are shown in Table 1 below.

### 2.2. Measures

In this study, the participants were asked to complete a demographic questionnaire (i.e., sex, age, school year, type of sports, and injury status) and a series of different questionnaires: (1) The short version of the Sport Climate Questionnaire (SCQ; [29]), (2) Controlling Coaching Behaviors (CCBS; Bartholomew, [27]), Sport Anxiety Scale-2 (SAS-2; [18]), and Athlete Burnout Questionnaire (ABQ; [12]). 

The SCQ and CCBS were used to measure athletes’ perception of their coaches’ coaching behaviors. First, the SCQ was used to assess the perceived autonomy-supportive coaching behaviors. The SCQ contains 6 items (e.g., “I feel that my coach provides me choices and options’’) measured on a 5-point Likert scale from 1 (strongly disagree) to 7 (strongly agree). The CCBS was used to assess athletes’ perception of controlling coaching behaviors. The CCBS is composed of four subscales: controlling use of rewards (“my coach tries to motivate me by promising to reward me if I do well”), negative conditional regard (“my coach is less friendly with me if I don’t make the effort to see things his/her way”), intimidation (“my coach shouts at me in front of others to make me do certain things”), and excessive personal control (“my coach expects my whole life to center on my sport participation”). The 15-item scale is measured on a 7-point Likert scale from 1 (strongly disagree) to 7 (strongly agree). The internal consistencies of the four subscales were acceptable (i.e., Cronbach’s alpha coefficients ranges from 0.83 to 0.29). The Korean version of SCQ and CCBS used in this study were reported to have acceptable reliability and validity [30]. 

The SAS-2 was used to measure athletes’ anxiety response tendencies to competitive sport situations, and it consists of 15 items, with five items on each of 3 subscales: worry as cognitive anxiety (e.g., “I worry that I won’t play well”), somatic anxiety (e.g., “My muscles feel tight because I am nervous”), and concentration disruption (e.g., “It is hard for me to focus on what I am supposed to do”). Each item is measured on a 4-point Likert scale from 1 (not at all) to 4 (very much so). The SAS-2 was reported to have acceptable reliability and factorial validity across samples in different age groups. Cho and his colleagues adopted the SAS-2 and examined its reliability and validity. They reported that the Korean version of SAS-2 had acceptable reliability and validity [31].

The ABQ was used to measure symptoms of athlete burnout. The ABQ is a sport-specific adaptation of the Maslach Burnout Inventory [32]. The ABQ is composed of three subscales: physical and emotional exhaustion (“I feel so tired from my training that I have trouble finding energy to do other things”), reduced sense of accomplishment (“I am not achieving much in my sport”), and sport devaluation (“the effort I spend in my sport would be better spent doing other things”). Each subscale contains 5 items measured on a 5-point Likert scale ranging from 1 (almost never) to 5 (always). According to Raedeke and Smith [1], burnout scores about 3 or above represent relatively high burnout in athletes. The ABQ has widely been used is the most widely used questionnaire with acceptable psychometric properties to measure burnout syndrome in the athlete population [33]. Choi and his colleagues adapted the ABQ and developed the Korean version of the ABQ [34]. They reported that the Korean version also had good reliability and validity. 

### 2.3. Procedures and Research Design

After the Institutional Review Board (IRB) approval was obtained, the authors contacted coaches of collegiate sport teams, explained the purpose of this study, and asked if they would allow the authors to visit their practice sites. With coaches’ permission, the authors visited coaches before their practice sessions started. Coaches brought the authors to their practice sites, introduced them to athletes, and left the sites. The authors informed the athletes of the study aims and explained that their participation was voluntary and anonymous and that they could withdraw from the participation without any penalty. They were also told to ask any questions before completing the series of questionnaires. Following Vandenbroucke et al.’s guidelines [35], a cross-sectional research design was employed for this study, and thus the data was collected only once. 

### 2.4. Statistical Analysis

Among 408 collegiate athletes with informed consent, 376 athletes completed the series of questionnaires (the response rate was 92.15%); however, 2 athletes withdrew, and 6 athletes did not answer many of the questions in the questionnaires. Therefore, the data completed by 368 athletes were analyzed for this study. 

Descriptive statistics, univariate skewness, univariate kurtosis, and correlations were calculated using the Statistical Package of the Social Sciences (SPSS 25.0, IBM, Chicago, IL, USA). The cut-off criteria of the univariate normality assumption were absolute values of 2 for skewness, and 7 for kurtosis [35]. Additionally, M*plus* 7 was used to conduct the structural equation modeling (SEM) to examine the full structural model. In congruence with previous studies [36,37], parceling strategies were used to increase the stability of the parameter estimates in the structural equation modeling procedures. First, construct-specific parcels were created for the CCBS, SAS-2, and ABQ. That is, the subscales that were in the three questionnaires were used as indicators. For example, physical and emotional exhaustion was used as one of three indicators for the ABQ. Second, item parcels were created for the SCQ. For items to construct balance, Little et al. [38] suggested that higher loaded items should be matched lower loaded items. Following Little et al.’s suggestion, within a subscale, a stronger loading item was paired with a weaker loading item. 

Anderson and Gerbing’s two-step approach [39] was utilized to evaluate the full structural model including the measurement model and structural model. The measurement model was conducted to examine the relationship between latent variables and their indicator variables, whereas the structural model was tested to evaluate the causal relationships between latent variables [40]. For the mediation effect analysis, 2000 bootstrap samples were requested [41]. With the chi-square (χ^2^) test, comparative fit index (CFI), and Tucker-Lewis index (TLI), standardized root mean square residual (SRMR), and root mean square error of approximation (RMSEA) were compared for evaluating the overall fit of the model. Previous studies suggest that the values of RMSEA below 0.08 [42], SRMR below 0.08, and CFI and TLI above 0.95 [43] were acceptable. construct reliability (CR) and average variance extracted (AVE) were calculated for convergent validity. The values of the CR suggested that the cut-off points of the CR and AVE values were 0.7 and 0.5, respectively [40]. 

## 3. Results

### 3.1. Descriptive Statistics

Table 2 shows the mean, standard deviation, skewness, kurtosis, and correlations of the scale composite scores. The overall values of skewness and kurtosis ranged from −0.11 to 0.27 and from −0.44 to −0.04, respectively, and, thus, the univariate normality was supported in that the absolute value of each item’s skewness was below 2 and kurtosis was below 7. The composite score correlation analyses showed significant correlations between variables except between autonomy-supportive coaching behaviors and competitive trait anxiety. 

Table 3 shows the correlation, mean, and standard deviation of the subscales used in this study. As mentioned, because we employed the construct-specific parceling strategy, each subscale was treated as an indicator in a scale except SCQ. 

### 3.2. Measurement Model

The fit indices for the measurement model were χ^2^(48) = 67.23 (*p* < 0.05), CFI = 0.99, TLI = 0.98, SRMR = 0.04, and RMSEA = 0.03 with 90% CI [0.01, 0.05]. The overall fit of the measurement model was acceptable. Standardized factor loading values of all items within the measurement model ranged from 0.58 to 0.94. The values of the CR ranging from 0.80 to 0.93 and AVE values ranging from 0.57 to 0.80 were above the suggested cutoff points of 0.07 and 0.05, respectively [26]. The values of standardized factor loading, CR, and AVE provided evidence for satisfactory convergent validity and internal consistency. 

### 3.3. Structural Model

**Figure 1:**
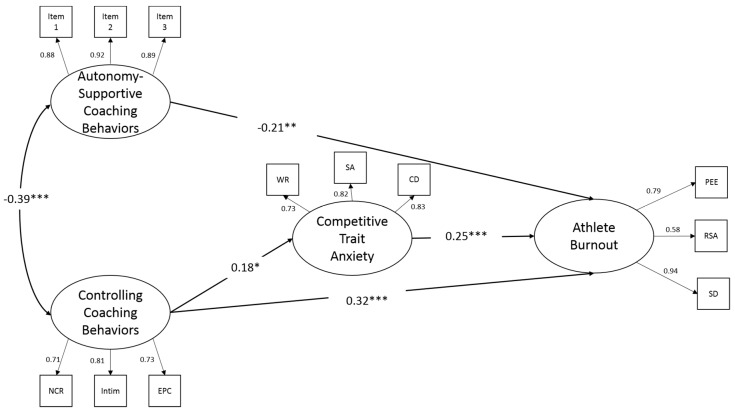
Structural equation model with standardized estimates in the relations between perceived coaching behaviors, competitive trait anxiety, and athlete burnout. Only significant pats are presented. Notes: * Significant at level *p* < 0.05, ** Significant at level *p* < 0.005, and *** Significant at level *p* < 0.001. NCR-negative conditional regard; Intim-intimidation; EPC-excessive personal control; WR-worry; SA-somatic anxiety; CD-concentration disruption; PEE-physical and emotional exhaustion; RSA-reduced sense of accomplishment; SD-sport devaluation.

We conducted path analyses of the structural model. Figure 1 shows the results of the path coefficients among the subscales. The fit indices for the structural model were χ^2^(48) = 67.23 (*p* < 0.05), CFI = 0.99, TLI = 0.98, SRMR= 0.03, and RMSEA = 0.03 with 90% CI [0.01, 0.05]. The overall model fit of the structural model was acceptable. Autonomy-supportive coaching was negatively related to athlete burnout (*β* = −0.21, *p* < 0.05), whereas controlling coaching and competitive anxiety were positively related to athlete burnout (*β* = 0.32, *p* < 0.01 and 0.25, *p* < 0.01, respectively). Intriguingly, only controlling coaching was significantly related to competitive trait anxiety (*β* = 0.18, *p* < 0.05). The bootstrapping results indicated the indirect path from controlling coaching to athlete burnout via competitive anxiety was significant (*β* = 0.05, *p* < 0.05).

## 4. Discussion

The purpose of this study was to examine the relationship between athletes’ perception of their coaches’ coaching behaviors, competitive trait anxiety, and burnout. Specifically, we aimed to investigate the medication effects of competitive trait anxiety on the relationship between perceived coaching behaviors and athlete burnout. 

First, consistent with previous studies, trait anxiety in athletes had a significant correlation with athlete burnout (*r* = 0.25, *p* < 0.01), as well as significant pathways (*β* = 0.25, *p* < 0.05). This finding confirmed that trait anxiety caused by chronic stress anxiety is an antecedent of burnout in athletes [14,15,16,19]. All three components of trait anxiety were significantly related to physical and emotional exhaustion and sport devaluation, but intriguingly there was no significant correlation between all three trait anxiety components and a reduced sense of accomplishment. This finding is inconsistent with previous studies [15,19,29,43], showing that all three components of trait anxiety were significantly related to accomplishment as well as the other two dimensions of athlete burnout and, thus, more trait-anxious athletes felt less personal accomplishment. 

The results of the correlations for the composite scores indicated that controlling coaching behaviors were significantly related to athlete’s competitive trait anxiety (*r* = 0.14, *p* < 0.01), whereas autonomy-supportive coaching behaviors were not significantly related to trait anxiety (*r* = −0.03, *p* = 0.57). The structural equation modeling results also showed a significant positive pathway from controlling coaching behaviors to trait anxiety (*β* = 0.18, *p* < 0.05), but not from autonomy-supportive coaching behaviors to trait anxiety (*β* = 0.03, *p* = 0.66). The current findings were consistent with Baker et al.’s findings [24] that negative coaching behavior was the strongest predictor of trait anxiety in athletes; however, it is surprising that autonomy-supportive coaching behaviors were not predictive of anxiety and consistent with previous studies. As Vealey et al. [15] and Baker et al. [24] explained, this unexpected finding might be due to the different scales used to measure perceived coaching behaviors. The core construct of both measures used in this study heavily relies on self-determined motivation, whereas other studies used measures based on leadership and other constructs. Additionally, some components in the controlling coaching behavior scale contain similar or the same constructs as other scales used in previous studies. For example, intimidation (“My coach shouts at me in front of others to make me do certain things”) used in this study is similar to the construct of negative rapport (“uses fear” and “yells when angry”) [24]. It may also be due to the notion that the unidimensional construct of the autonomy-supportive coaching behavior questionnaire was not able to measure various positive coaching behaviors.

Excessive personal control was significantly related to all three subscales in trait anxiety, and intimidation was related to two (i.e., somatic anxiety and concentration disruption), but negative conditional regard did not have significant correlations with any anxiety subscales. Overall, these findings support the notion that “certain coaching behaviors are better predictors of sport anxiety” [24] (p.116). As shown in Table 2, excessive personal control had stronger relationships with three more trait anxiety components than intimidation and negative conditional regard had. The participants in this study were collegiate athletes, and they have many different kinds of stress, such as athletic stress and academic stress. When they felt that their coaches tried to control not only sport-related matters but also their life and free time outside of sports, athletes can perceive these behaviors as “over-intrusive behaviors” [27] (p.197) which can also be a strong precursor to increase anxiety levels in athletes. 

Both autonomy-supportive and controlling coaching behaviors were significantly related to athlete burnout (*r* = −0.33, *p* < 0.01 and *r* = 0.41, *p* < 0.01, respectively). All components of controlling coaching behaviors were also significantly correlated with all three dimensions of athlete burnout. Excessive personal control had the strongest correlations with the three athlete burnout dimensions. The structural equation modeling result indicated that both autonomy-supportive and controlling coaching behaviors had significant pathways to athlete burnout (*β* = −0.21, *p* < 0.05 and *β* = 0.32, *p* < 0.001, respectively). The findings support previous research that indicated perceived coaching behaviors was the main precursor of burnout in collegiate athletes [15,16,23,24,25,26,44]. The bootstrapping results indicated a significant indirect pathway from controlling coaching to athlete burnout via competitive trait anxiety, and this is inconsistent with Vealey et al.’s findings that perceived coaching behaviors had only a direct effect to burnout but did not have indirect effects on burnout via trait anxiety [16]. As mentioned, this may be due to the fact that each study used different scales. 

There are several limitations to generalize the current findings. First, in this study, a cross-sectional design was used to collect the data. The cross-sectional approach is limited in providing clear causal relationships between variables. Athlete burnout may change before, during, and after seasons. It is possible that coaches provide more autonomy-supportive coaching before the season and then more controlling coaching after a season starts. Therefore, future research using a longitudinal approach is needed to examine how perceived coaching behaviors affect athlete burnout and to test the mediating effects of trait anxiety on the relationship between perceived coaching behaviors and athlete burnout. Second, due to relatively small samples, we could not conduct invariance analyses across sex (i.e., males vs. females) and types of sports (i.e., individual vs. team sports). There are usually more numbers of athletes in team sport than in individual sports (e.g., soccer team vs. golf team). Due to bigger numbers in team sports, a coach in a team sport might employ more controlling coaching to manage the number of athletes compared to a coach in an individual sport. Therefore, future research needs to have equal or similar sample sizes in groups and at least 200 participants in each group to conduct invariance analyses. As previous researchers pointed out [15,24], there are various scales to measure athletes’ perceptions of their coaches’ behaviors. This caused inconsistent findings in the relationship between perceived coaching behaviors and anxiety. A new questionnaire development to measure perceived coaching behaviors more precisely or a re-evaluation of the current scales should be conducted. This study tested one interpersonal factor (perceived coaching behaviors) and one intrapersonal factor (trait anxiety) that could influence athlete burnout. Other previous studies examined the relationship between other interpersonal factors (e.g., parent–athlete relationship and peer support) and/or intrapersonal factors (e.g., perfectionism) and athlete burnout. Future research should investigate simultaneously the relationship between athlete burnout and multi intrapersonal and interpersonal factors in order to clearly understand the antecedents of burnout and their relationships.

## 5. Conclusions

The results supported the notion that athletes’ perceptions of their coaches’ behaviors were of importance to understanding trait anxiety and burnout in athletes, especially in competitive sports. The present findings provide practical information for coaches, educators in sports, and consultants. Coaching behaviors are not dichotomous. That is, a coach can provide autonomy-supportive coaching and at the same time controlling coaching, and both perceived coaching behaviors can affect athletes’ performance and psychological well-being. Given that controlling coaching behaviors affected trait anxiety and, in turn, burnout, the findings of this study suggest that should coaches provide less controlling coaching to reduce anxiety and burnout in athletes. Because intimidation and excessive personal control were related to both anxiety and burnout, education for coaches about communication and coaching must be provided. Practical consultants should regularly provide relaxation, mediation, and mindfulness intervention for athletes to decrease somatic anxiety and increase concentration, and in turn to decrease burnout. Competitiveness is a core component of sports, and competitive environments cannot be removed from sports settings. Cognitive behavioral intervention for athletes must be provided to manage their stress and decrease burnout [45]. 

## Figures and Tables

**Table 1:** General characteristics of the participants (*n* = 368).

Characteristics	Category	Frequency	Present
Sex	Male	280	78.3
	Female	80	21.7
Age	20	111	30.2
	21	126	34.2
	22	79	21.5
	23	47	12.8
	24 or older	5	1.4
School year	Freshmen	111	30.2
	Sophomores	126	34.2
	Juniors	79	21.5
	Seniors	47	12.8
	Graduate school	5	1.4
Type of Sports	Track and field	27	7.3
	Weightlifting	16	4.3
	Swimming	42	11.4
	Gymnastics	18	4.9
	Golf	36	9.8
	Basketball	45	12.2
	Taekwondo	67	18.2
	Baseball	35	9.5
	Judo	41	11.1
	Soccer	41	11.1

**Table 2:** Mean (*M*), standard deviation (*SD*), skewness, kurtosis, and correlations of scale composite scores.

Scale	*M*	*SD*	Skewness	Kurtosis	1	2	3
1. SCQ	4.62	1.23	−0.04	−0.12	-		
2. CCBS	3.21	1.11	−0.07	−0.47	−0.35 **	-	
3. SAS-2	1.93	0.59	0.50	−0.06	−0.03	0.14 **	-
4. ABQ	2.70	0.67	−0.12	−0.44	−0.33 **	0.41 **	0.25 **

Note: Sport Climate Questionnaire (SCQ) for autonomy-supportive coaching behaviors, Controlling Coaching Behaviors (CCBS) for controlling coaching behaviors, Sport Anxiety Scale-2 (SAS-2) for competitive trait anxiety, and Athlete Burnout Questionnaire (ABQ) for athlete burnout. ** *p* < 0.01, two-tailed test.

**Table 3:** Correlation, Mean (*M*), and standard deviation (*SD*) of subscales of CCBS, SAS-2, ABQ, and total SCQ.

	1	2	3	4	5	6	7	8	9	10
1										
2	0.60 *									
3	0.51 **	0.59 **								
4	0.03	0.11 *	0.14 **							
5	0.05	0.07	0.12 *	0.60 **						
6	0.09	0.12 *	0.18 **	0.68 **	0.60 **					
7	0.20 **	0.25 **	0.31 **	0.26 **	0.27 **	0.25 **				
8	0.28 **	0.28 **	0.34 **	0.07	0.05	0.07	0.42 **			
9	0.27 **	0.31 **	0.37 **	0.21 **	0.21 **	0.26 **	0.55 **	0.74 **		
10	−0.26 **	−0.31 **	−0.30 **	−0.01	−0.00	−0.08	−0.29 **	−0.22 **	−0.31 **	
*M*	3.57	3.19	2.88	1.82	2.23	1.76	2.54	2.97	2.59	4.62
*SD*	1.19	1.31	1.46	0.67	0.77	0.60	0.75	0.73	0.88	1.23

Note: 1. negative conditional regard; 2. intimidation; 3. excessive personal control; 4. somatic anxiety; 5. worry; 6. concentration disruption; 7. physical and emotional exhaustion; 8. Reduced sense of accomplishment; 9. sport devaluation; 10. autonomy-supportive coaching behaviors. Subscales of CCBS are 1, 2, and 3. Subscales of SAS-2 are 4, 5, and 6. Subscales of ABQ are 7, 8, and 9. * *p* < 0.05, two-tailed test. ** *p* < 0.01, two-tailed test.
